# Eczéma de contact bulleux suite à un tatouage au henné noir

**DOI:** 10.11604/pamj.2013.15.148.3261

**Published:** 2013-08-27

**Authors:** Karima Chakir, Badreddine Hassam

**Affiliations:** 1 Service de Dermatologie et Vénérologie, CHU Ibn SINA, Rabat, Maroc

**Keywords:** Eczéma de contact, tatouage, henné, Contact dermatitis, tattoo, henna

## Image en médicine

Le henné ou Lawsonia inermis est utilisé depuis l'antiquité pour la coloration des cheveux, des mains et des pieds des femmes orientales. Les eczémas de contact au henné pur sont très rares, ils sont dus le plus souvent à des additifs tels les huiles parfumées ou la paraphénylène diamine (PPD). Nous rapportons le cas d'une jeune femme qui avait présenté un eczéma de contact au henné noir; 24 heures après son application elle a eu une sensation de cuisson et de prurit suivie de l'apparition de lésions vésiculobulleuses augmentant rapidement de volume pour devenir bulleuse à contenu clair suivant intimement le dessin du tatouage; la patiente a été mise sous dermocorticoïdes classe II après avoir percé les bulles par une aiguille stérile; l’évolution était bonne. Actuellement, le henné est très en vogue dans les pays occidentaux. La PPD est ajoutée pour diminuer le temps de fixation ou pour obtenir une coloration plus foncée. Elle peut entraîner de graves réactions systémiques à type d'angio-œdème ou de choc anaphylactique. La réaction allergique la plus fréquente est la dermatite de contact. Une meilleure législation sur la pratique du tatouage temporaire et le contrôle des préparations ainsi qu'une information régulière annuelle du grand public sont indispensables.

**Figure 1:**
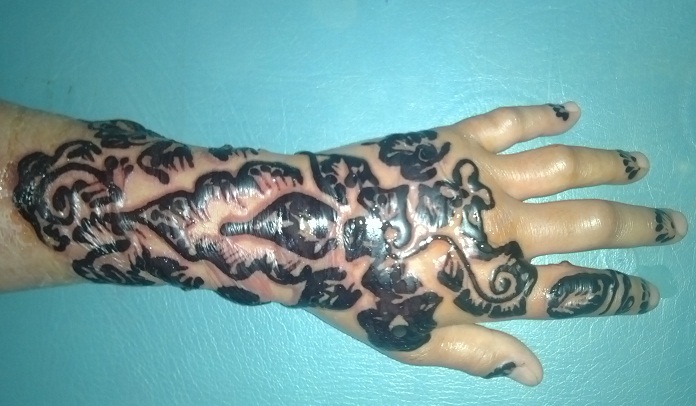
Eczéma de contact bulleux

